# Conformational Flexibility of the C-Terminal Region Influences Distal Active Site Residues Across the Tautomerase Superfamily

**DOI:** 10.3390/ijms252312617

**Published:** 2024-11-24

**Authors:** Christopher Argueta, Andrew Parkins, Georgios Pantouris

**Affiliations:** Department of Chemistry, University of the Pacific, Stockton, CA 95211, USA; c_argueta@u.pacific.edu (C.A.); a_parkins@u.pacific.edu (A.P.)

**Keywords:** Tautomerase superfamily (TSF), macrophage migration inhibitory factor (MIF), D-dopachrome tautomerase (D-DT), 5-carboxymethyl-2-hydroxymuconate isomerase (CHMI), cis-3-chloroacrylic acid dehalogenase (cis-CaaD), 4-oxalocrotonate tautomerase (4-OT), malonate semialdehyde decarboxylase (MSAD), protein dynamics, molecular dynamics (MD) simulations

## Abstract

Consisting of more than 11,000 members distributed over five families, the tautomerase superfamily (TSF) is a large collection of proteins with diverse biological functions. While much attention has been given to individual TSF enzymes, a majority remain structurally and functionally uncharacterized. Given its large size, studying a representative member of each family offers a viable approach for extracting mechanistic insights applicable to the entire superfamily. In this study, cis-3-chloroacrylic acid dehalogenase (cis-CaaD), 5-carboxymethyl-2-hydroxymuconate isomerase (CHMI), malonate semialdehyde decarboxylase (MSAD), and 4-oxalocrotonate tautomerase (4-OT) were referenced against the well-studied macrophage migration inhibitory factor (MIF) and D-dopachrome tautomerase (D-DT) using triplicate 1 μs molecular dynamics (MD) simulations for a total of 18 μs. Through root mean square fluctuation (RMSF) measurements, correlation analyses, and comparisons to previous crystallographic structures, we reveal key mechanistic insights that promote the understanding of the catalytic activities in TSF. Collectively, our findings from these functionally diverse TSF proteins provide key information on allosteric coupling, long-range intra- and inter-subunit communications as well as structure–activity relationships that enable new studies in the superfamily.

## 1. Introduction

The tautomerase superfamily (TSF) is a ubiquitous group of proteins found across all domains of life, including Archaea, Bacteria, and Eukarya [1]. This superfamily is composed of five families, each represented by a founding protein: macrophage migration inhibitory factor (MIF), cis-3-chloroacrylic acid dehalogenase (cis-CaaD), 5-carboxymethyl-2-hydroxymuconate isomerase (CHMI), malonate semialdehyde decarboxylase (MSAD), and 4-oxalocrotonate tautomerase (4-OT). Members of the TSF are involved in a variety of biological processes and possess diverse catalytic activities across a range of substrates [1]. Despite their functional versatility, TSF proteins are characterized by a shared β-α-β structural motif and a conserved N-terminal proline (P1) that serves as either a catalytic base or acid [2,3]. A striking exception to this rule is ~3% of TSF proteins (346 out 11,395) that lack P1, yet they are clearly part of this superfamily [1]. Oligomeric organization of TSF proteins leads to the formation of functional dimers [3], trimers [4,5], and hexamers [6] with a solvent channel at the center of the assembly. Studies have shown that ordered water molecules found within the channel play a functional role in influencing the catalytic activity of the protein carrier [3,7].

Regardless of the knowledge we have gained thus far, the majority of TSF enzymes remain structurally and functionally uncharacterized. Only select members of this superfamily, such as MIF and D-DT, have attracted a lot of attention due to their pleiotropic functionalities in human pathophysiology [8,9,10,11,12,13]. For their structural [14,15,16,17,18] and functional [18,19,20,21] characterization, small molecule modulators discovered through the shared tautomerase activity [22,23] were used as experimental tools. Other TSF enzymes that have been characterized, but are not found in humans, are the founding members of the remaining four families: 4-OT, CHMI, cis-CaaD, and MSAD.

4-OT is a small protein consisting of just 62 amino acids [24] that catalyzes the isomerization of unsaturated alpha-keto acids [25]. Its homohexameric biological assembly, drastically differs from the typical trimeric structures of CHMI [26], cis-CaaD [27], and MSAD [28]. In 4-OT, P1 facilitates catalysis by abstracting a proton from the substrate (e.g., 4-oxalocrotonate) thus, serving as a catalytic base [3]. Protein crystallography, nuclear magnetic resonance (NMR), and biochemical assays exposed R11, R39, and F50 as additional key active site residues [3].

With two fused β-α-β units, CHMI is approximately twice as long as monomeric 4-OT. Its commonly known substrate is 5-(carboxymethyl)-2-hydroxymuconate (CHM) [26] and the catalytic mechanism involves P1 acting as a general base [2]. Similar to 4-OT, the active site environment of CHMI includes two arginine residues (R40 and R71) that serve a key role in catalysis [2]. In *Escherichia coli C*, CHMI is expressed along with other enzymes as part of the homoprotocatechuate (hpc) pathway to generate intermediates of the citric acid cycle enabling the production of carbon and energy from aromatic amino acids catabolism [29].

The TSF enzyme cis-CaaD is known to catalyze the hydrolytic dehalogenation of cis-3-chloroacrylic acid by converting it into malonate semialdehyde. The conserved P1 exhibits a p*K*_a_ of ~9.3, allowing it to function as a general acid [30], while R70 and R73 are involved in substrate binding and stabilization [27]. H28 and Y103 were identified in crystallographic studies as potential key players in the enzyme’s activity by contributing to substrate specificity as well as catalytic mechanism.

MSAD is involved in decarboxylation reactions such as the conversion of malonate semialdehyde into acetaldehyde and carbon dioxide. Similar to cis-CaaD, the catalytic residue P1 of MSAD functions as a general acid (p*K_a_* of ~9.2) [31,32], while residues D37, R73, and R75 aide in the active site catalysis [28]. MSAD exhibits promiscuous hydratase activity, in addition to its well-known decarboxylase one. This new functionality was confirmed through inhibition studies using 3-bromo- and 3-chloropropiolate, the first identified MSAD inhibitors [33].

Because the dynamic profiles of MIF and D-DT were successfully mapped with 1 μs molecular dynamics (MD) simulations [16,34] that yielded not only structural but also functional insights to these pleiotropic proteins, we applied a similar approach on CHMI, cis-CaaD, MSAD, and 4-OT. While the dynamic profiles of these four proteins is vastly unknown, our goal was to extract mechanistic insights that would be applicable not only to these proteins but also to the entire superfamily. Our findings on the four proteins highlight the key role of long-range intra- and inter-subunit communication that corroborate the dynamic correlation of the C-terminal region with active site residues. Such findings offer a deeper understanding of the catalytic mechanisms and functional activities of TSF, preparing the ground for additional studies of uncharacterized superfamily members.

## 2. Results

### 2.1. Crystallographic Analysis of TSF Representative Members Reveals Noticeable Differences in the C-Terminal Region

Previously published studies on the TSF human members, MIF and D-DT, showed that the C-terminal region has a multi-tiered role associated with the structure and function of these proteins [14,16,17,34,35,36]. Having these two proteins as our benchmark, we probed for broader insights into this superfamily. While interrogation of over 11,000 proteins is unrealistic, we selected representative members based on a previously published sequence similarity network (SSN) analysis that partitioned the TSF proteins into five families; MIF, cis-CaaD, MSAD, CHMI, and 4-OT [1].

**Figure 1:**
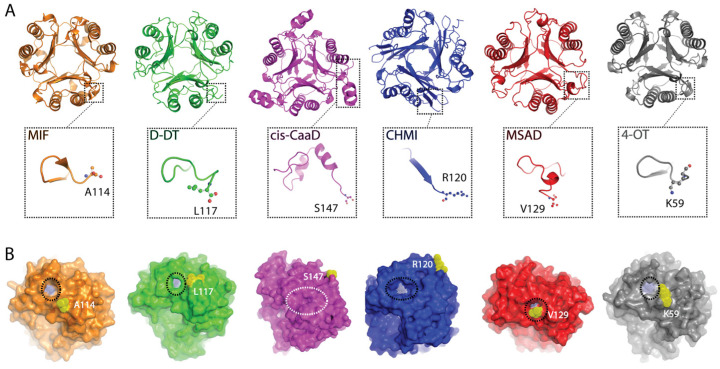
Structural homology between the TSF members. (**A**) The biological assemblies of MIF (orange—PDB:3DJH), D-DT (green—PDB:1DPT), cis-CaaD (magenta—PDB:2FLZ), CHMI (blue—PDB:3E6Q), MSAD (red—PDB:2AAG), and 4-OT (grey—PDB:1OTF) demonstrate an overall satisfactory structural homology, with some noticeable differences in the C-terminal region (dashed boxes). The C-terminus residue for each protein is shown in ball and sticks representation. (**B**) Surface analysis focused on the area around the active site opening (black dashed circles) shows the distance between P1 and the C-terminus residue for each protein. P1 and the C-terminus residue are illustrated with light blue and yellow dots, respectively. In the case of cis-CaaD, the active site opening (white dashed circle) is not visible as it is blocked by the β8/α3 loop of C-terminus.

A multiple sequence alignment was performed utilizing the amino acid sequences of human MIF, human D-DT, *Coryneform bacterium* cis-CaaD, *Pseudomonas aeruginosa* CHMI, *Pseudomonas pavonaceae* MSAD, and *Pseudomonas* sp. (strain CF600) 4-OT (Figure S1). Considering the available crystal structures, deposited in protein data bank (PDB), our protein selection was made to ensure that the structural and dynamic properties of each protein family will be accurately analyzed. While the amino acid sequences come from different organism sources, the generated findings ensures broader applicability which is critical considering the size of this superfamily (>11,000). The highest sequence identity (SeqID) of 34.2% between any protein pair was obtained between MIF and D-DT (Table S1). The remaining protein pairs yielded values under 30%, with MSAD exhibiting similar SeqIDs with 4-OT (25.8%), cis-CaaD (25.0%), and CHMI (24.7%). Despite the low amino acid sequence identity, the six proteins demonstrate an overall satisfactory structural homology at the quaternary level, yet with diverse C-terminal segments (Figure 1A). Specifically, key differences were noted in the length (Figure S1) and secondary structure organization of the C-terminal tail (Figure 1A) as well as the position of the C-terminus in relation to the active site pocket (Figure 1B). For MIF, D-DT, MSAD, and 4-OT, the C-terminus is proximal to the active site opening, whereas in the cases of cis-CaaD and CHMI, the C-terminus is distal from it (Figure 1B). Interestingly, the active site opening of cis-CaaD is completely blocked by the β8/α3 loop, which is part of the C-terminal tail.

### 2.2. Root Mean Square Fluctuation (RMSF) Analysis Across the Target Proteins Demonstrates Diverse Dynamic Profiles

**Figure 2:**
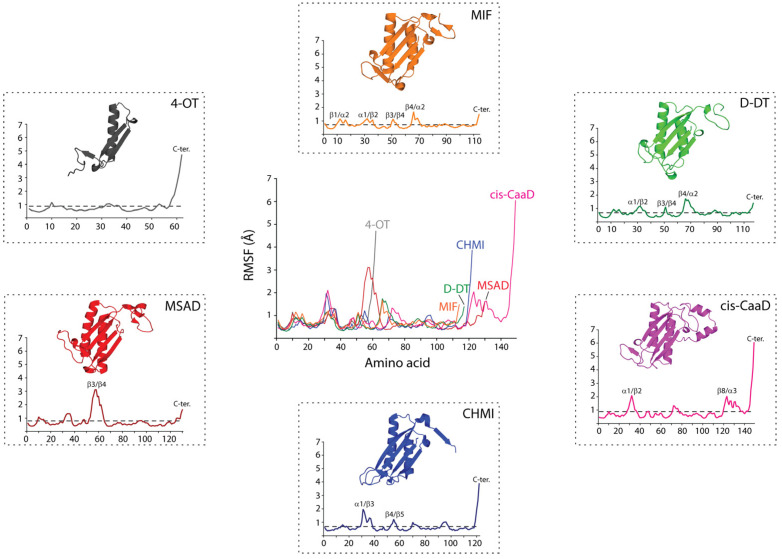
Average RMSF profiles of TSF proteins. The average fluctuations of protein residues (x-axis) were derived from three independent runs and monitored over the course of 1 μs MD simulations. The RMSF magnitude (y-axis) is expressed in angstroms (Å). Regions with fluctuation values greater than 1σ of the mean RMSF value (dashed lines) are marked using the secondary structure features of each protein. The MIF (orange), D-DT (green), cis-CaaD (magenta), CHMI (blue), MSAD (red), and 4-OT (grey) monomers are also shown. For comparison, the overlay of all six profiles is shown at the center.

Upon identifying these structural differences in the C-terminal region, we performed MD simulations and analyzed the RMSF profiles of the six proteins (Figure 2 and Figure S2). 1 μs trajectories were considered suitable for investigating the C-terminal motions [37], while each calculation was repeated in triplicate. Globally, the six proteins demonstrated similar fluctuations with an average RMSF value of 0.71 ± 0.2 Å, 0.69 ± 0.3 Å, 0.88 ± 0.7 Å, 0.67 ± 0.5 Å, 0.79 ± 0.5 Å, and 0.87 ± 0.7 Å for MIF, D-DT, cis-CaaD, CHMI, MSAD, and 4-OT, respectively (Table S2, Figure S2). To confirm the accuracy of our approach, we compared our findings with previously published RMSF values obtained from 1 μs trajectories. In the absence of any data for the bacterial proteins, we considered only data derived from the two human proteins, MIF and D-DT. The previously published RMSF values of 0.90 Å [34] and 0.70 Å [16], for MIF and D-DT, respectively, are in agreement with the findings of this study (0.71 ± 0.2 Å (MIF) and 0.69 ± 0.3 Å (D-DT)). Having confirmed the accuracy of our calculation, we performed an in-depth analysis of the RMSF results focusing on the poorly studied bacterial proteins. For clarity, the secondary structure features of cis-CaaD, CHMI, MSAD monomers, and 4-OT dimer are provided (Figure S3).

The high similarity between the RMSF profiles of MIF and D-DT is apparent even upon a brief examination. However, each of the four bacterial proteins exhibits characteristic RMSF patterns that suggest unique intra- and inter-subunit communication pathways (Figure 2). Notably, for the two human proteins, the highest RMSF value of any region does not surpass 2 Å. Meanwhile, cis-CaaD, CHMI, MSAD, and 4-OT enclose one highly flexible region with an RMSF value far exceeding 2 Å. For CHMI, cis-CaaD, and 4-OT, this region is located in the C-terminal, whereas in MSAD, the β3/β4 loop is highly flexible. The reduced flexibility noted in the C-terminal residues of MSAD more closely resembles what is observed in MIF and D-DT (Figure 2). From a structural point of view, the high flexibility of cis-CaaD in the C-terminal tail, is of a great functional interest as it influences the mobility of β8/α3 loop, which in turn blocks the opening to the active site (Figure 1B).

Regions with fluctuation values greater than 1σ of the mean RMSF value were marked using the secondary structure features of each protein (Figure 2 and Figure S3). Our findings show that the human proteins have more regions with statistically significant RMSF values in comparison to the bacterial ones. This finding is not attributed to the enhanced flexibility of MIF and D-DT, but rather, it is explained by the presence of highly flexible regions within the four bacterial proteins. For this reason, we overlayed the six profiles and examined their fluctuation features region-by-region (Figure 2). The varying secondary structural features and length of the six proteins account for the differences seen in the overlayed RMSF illustration. Despite this, a similar fluctuation pattern with values above the average was noted in the α1/β2 loop (corresponding to the α1/β3 loop of CHMI). This loop is located in the active site cavity of all the proteins and is specifically adjacent to the catalytic residue P1 (Figure 2 and Figure S4). With reference to the better studied human proteins, MIF and D-DT, this loop includes residues that are important for catalysis and ligand binding [17,18,22,38]. Excluding MSAD, fluctuation above the mean value was also noted for residues found in the β4/α2 loop of MIF/D-DT and the corresponding β5/α2 and β1/α1 loops of cis-CaaD/CHMI and 4-OT, respectively. This loop, which is also located next to the catalytic residue P1, harbors an active site residue: I64 in MIF and D-DT, R11 in 4-OT, R71 in CHMI, R73 in cis-CaaD, and R75 in MSAD. In all proteins, fluctuation of the C-terminal region was found to be significant, surpassing 1σ of the mean RMSF value.

### 2.3. Correlation Plots Expose Communication Pathways with Mechanistic Interest

**Figure 3:**
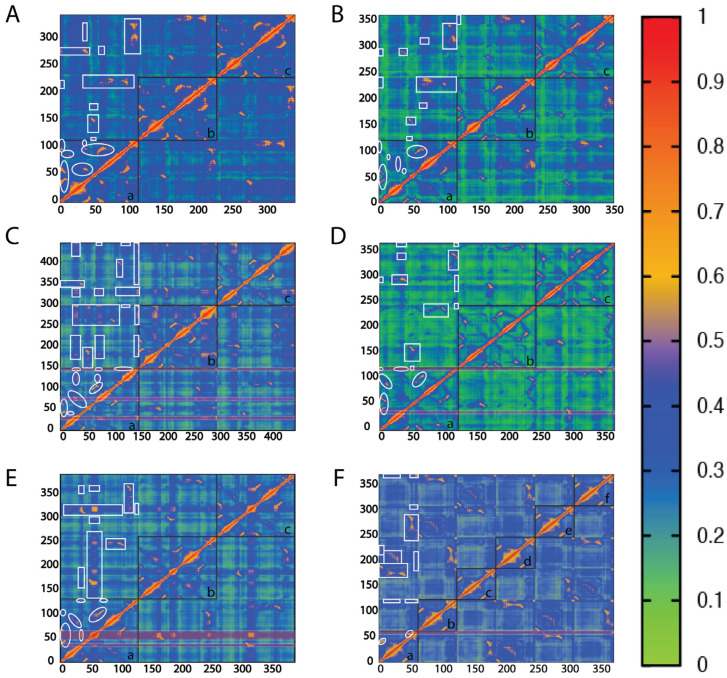
Correlation analysis of the TSF members. Generalized correlated Cα motions for (**A**) MIF, (**B**) D-DT, (**C**) cis-CaaD, (**D**) CHMI, (**E**) MSAD, and (**F**) 4-OT are provided side-by-side for comparison. Each correlation plot represents the average analysis derived from three 1 μs trajectories. Correlation coefficients range from 0 to 1, as shown in the heat map. Oval circles and boxes highlight intra-subunit and inter-subunit correlated regions, respectively. The black boxes (a–c) shown in the correlation plots of MIF, D-DT, cis-CaaD, CHMI, and MSAD demonstrate the three subunits of these proteins. The corresponding six subunits of 4-OT are shown by the black boxes (a–f). The Y- and X-axes show the residue numbers of each protein. Correlations possessing mechanistic interest are marked with horizontal rectangles filled with transparent red.

Correlation analyses of the Cα atoms were performed for the six proteins included in this study (Figure 3A–F). Similar to the RMSF analysis, MIF and D-DT (Figure 3A,B) were only used as controls to validate our data against previously published findings [16,34]. Once the reproducibility of our approach was confirmed, our attention shifted to the four bacterial proteins whose communication pathways are unknown (Figure 3C–F).

In all four proteins, the β1 strand is strongly correlated with the two adjacent strands of the monomeric β sheet, dimeric in the case of 4-OT. This finding is consistent with previously published observations of the two human proteins [16,34] and it reflects the fundamental role of the β sheet in correlated motions [39,40]. With cis-CaaD being a notable exception, we found another shared correlation between the first residue of β1 and the strand defining the solvent channel opening; β5 for MSAD, β7 for CHMI, and β2 of the adjacent monomer for 4-OT (Figure S3). This correlation is of great importance for the two human proteins as it was shown to modulate their catalytic activities via allosteric coupling [7,41].

**Figure 4:**
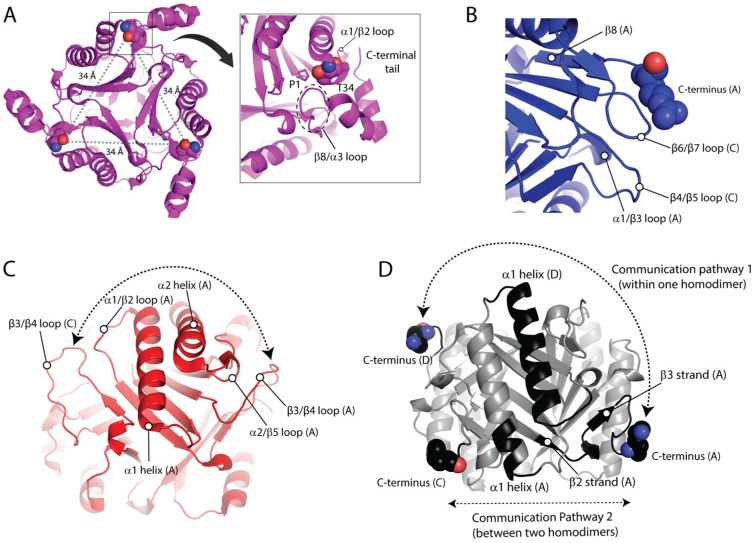
Correlations with mechanistic interest in cis-CaaD, CHMI, MSAD, and 4-OT. (**A**) T34 demonstrates high correlations with itself in all the subunits of cis-CaaD, despite the large distance of 34 Å between them. Intra-subunit correlations of the α1/β2 loop, on which T34 is situated, involve interactions with the C-terminal tail and the β8/α3 loop (dashed circles). P1 and T34 are shown as sticks and space filling representation, respectively. (**B**) The C-terminus (in space filling representation) of CHMI communicates with the α1/β3 loop through inter-subunit correlations that involve the β4/β5 and β6/β7 loops of the adjacent monomer. (**C**) The highly flexible β3/β4 loop of MSAD from subunit A is highly correlated with the β3/β4 loop of the adjacent subunit C. These correlated motions are mediated through intermediate communications that involve the α1/β2 loop, α1 helix, α2 helix, and α2/β5 loop of subunit A. (**D**) Within the same pseudo-monomer of 4-OT, the C-terminus (in space filling representation) of monomer A communicates with the C-terminus of monomer D through the α1/β2 loop, α1 and β1/α1 loops. Communication between the C-terminus of monomer A and monomer C, which is the subunit of the adjacent homodimer, occur in the order described via the β3 strand, β2/β3 loop, β2 strand, and α1 helix.

With the objective to discover mechanistic insights into cis-CaaD, CHMI, MSAD, and 4-OT, we probed characteristic communications for each protein. In cis-CaaD, T34 stands out as a key residue situated on the flexible α1/β2 loop (Figure 4A). Our analysis showed that this residue is strongly correlated with multiple domains across the biological assembly of cis-CaaD (Figure 3C). Within the same subunit, T34 communicates with the β8/α3 loop, the α3 helix, and the C-terminal tail, which are all found proximal to this residue (Figure 4A). Noteworthy, β8/α3 is the loop blocking accessibility to the active site pocket (Figure 1B). T34 of monomer A also forms inter-subunit communications with the α1/β2 loops of monomers B and C, including their T34 residues which are found ∼34 Å apart (Figure 4A). In addition, T34 of monomer A is correlated with the β8/α3 loop and the α3 helix of monomer B as well as the three C-termini of cis-CaaD. These communications take place via long-range inter-subunit crosstalk with the participation of β5/α2 and β6/β7 loops as well as segments of the α2 helix and the β6 strand (Figure S3). With the exception of the β5/α2 loop, the remaining regions are located in the subunit-subunit interface and enable communications with the adjacent monomer. These findings led to the conclusion that catalysis in cis-CaaD is a highly coordinated process, where T34 plays a key role via major conformational changes in the C-terminal region β8/α3 loop, α3 helix, and the C-terminal tail.

For CHMI, an interesting observation is that all the C-termini are correlated with one another, despite being situated ~47 Å apart (Figure 3D). These correlations are enabled through the participation of multiple regions, including the α1/β3 loop that is strongly linked with the C-terminus (Figure 4B). Interestingly, the β8 strand that is found just before the C-terminus packs against the adjacent subunit. When examining subunit A, we observe that the β8 strand of this subunit interacts with the β7 strand of subunit C, bringing the C-terminus of subunit A proximal to the β6/β7 loop of subunit C (Figure 4B). The β6/β7 loop of subunit C is highly correlated with the β4/β5 loop of the same subunit, as well as the α1/β3 loop of subunit A. These correlated motions initiate from the highly flexible C-terminal region of subunit A, propagate through the β6/β7 loop of subunit C, and continue into the β4/β5 loop of the same subunit, ultimately reaching the α1/β3 loop of subunit A (Figure 4B). The α1/β3 loop of CHMI is characterized as the second highest RMSF region, surpassed only by the C-terminus. The correlated motions travel up towards the ends of β5 and β7 and, ultimately, reach the C-terminal solvent channel opening. Residues located at the end of β7 and within the β7/β8 loop of subunit A are correlated with the same residues of subunit C due to proximity, effectively bridging the C-terminus of subunit A and the C-terminus of subunit C.

From the RMSF analysis (Figure 2), it was clearly shown that MSAD exhibits distinct dynamic characteristics from cis-CaaD, CHMI, and 4-OT. A unique aspect of MSAD is that the region with the highest RMSF value is not the C-terminus, as is typically observed in the other three bacterial proteins, but rather the loop between β3 and β4 strands (Figure 4C). The correlation analysis also highlights that the β3/β4 loop is a region of high mechanistic value forming multiple strong intra- and inter-subunit communications across the biological assembly of MSAD (Figure 3E). The α1/β2 loop also exhibits a significant number of correlations, second only to the β3/β4 loop. The communication network formed through the β3/β4 loop as well as the α1/β2 loop facilitate the correlated motions within MSAD. Further analysis showed that the β3/β4 loop from different subunits form strong correlations with each other through intermediate communications that involve α1/β2 loop, α1, α2 helices, and α2/β5 loop (Figure 4C).

In the case of 4-OT, the communication pathways found in the biological assembly are more complicated in comparison to the other three proteins due to the homohexameric structure. For comparative analysis with the other TSF proteins, a dimer of 4-OT effectively acts as a pseudo-monomer, thereby incorporating two C-terminal regions within one monomer (Figure S3). Monomers A&D, B&E, and C&F form three homodimers, each of which corresponds to a monomer of cis-CaaD, CHMI, and MSAD. Analysis of the correlation data showed that the six C-termini communicated with each other despite the long distances (the shortest determined at ~26 Å). The flexibility observed in the C-terminal residues of the protein (Figure 2) plays a key role in the intra- and inter-subunit communication pathways enabling cross-talking between the C-termini. Our findings demonstrate that the communication pathway between the two C-termini of a given homodimer (pseudo-monomer) differs from the corresponding pathways formed across two homodimers (Figure 4D). Using the homodimer A/D as an example and in the order described, the C-terminus of monomer A communicates with the C-terminus of monomer D through the α1/β2, α1 and β1/α1 loops (Figure 4D). Communication between the C-terminus of monomer A and monomer C, which is a subunit of the adjacent homodimer, occur through the β3 strand, β2/β3 loop, β2 strand, and α1 helix (Figure 4D).

### 2.4. Correlation Analyses Reveals Communications Between the C-Terminal Region and Active Site Residues

**Figure 5:**
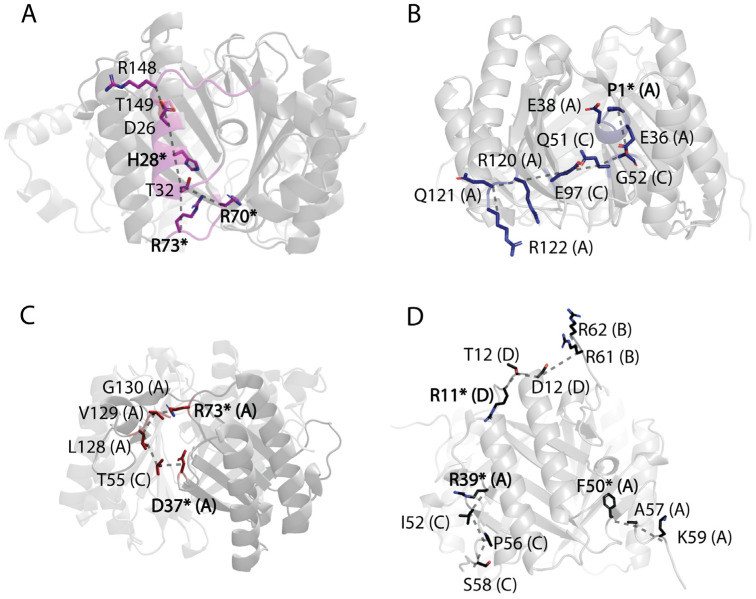
Communication pathways between the C-terminal region and active site residues. Correlation analyses of (**A**) cis-CaaD, (**B**) CHMI, (**C**) MSAD, and (**D**) 4-OT demonstrate that the C-terminal region and active site residues for each protein form distinct communication pathways (dashed grey lines). The residues involved in each pathway are shown as sticks. Bold residues with an asterisk indicate the active site residues. When multiple subunits (subunit A, B, etc.) are involved in the communication pathway, the letter of each subunit is indicated in parenthesis.

As previously described, communications between the C-terminal region and active site residues modulate the enzymatic activities of MIF and D-DT. Bearing this in mind, we utilized our correlation analyses to detect distinct communication pathways in cis-CaaD, CHMI, MSAD, and 4-OT. Among the six proteins of this study, cis-CaaD is the largest, featuring a highly flexible C-terminal region (Figure 2) and exhibiting high intramolecular correlation with the α1 helix (Figure 3C). The active site residue H28 lies on this helix, but the correlated motions continue through the α1 helix towards R70 and R73 (Figure 5A), which are also active site residues.

For CHMI, our findings demonstrate cross-talk between residues situated at the interface of two monomers that bridge the C-terminal region with the catalytic residue P1 (Figure 5B). Specifically, the C-terminal region of subunit A is correlated to P1 of the same subunit via the β6/β7 loop derived from subunit C. From the β6/β7 loop, the correlated motions continue towards the β4/β5 loop of subunit C, then onto the α1/β3 loop of subunit A to eventually reach P1 (Figure 5B).

In MSAD, the C-terminal region exhibits greater restraint; however, a sizable and profoundly dynamic region of the β3/β4 loop within one subunit is positioned between the C-terminal segment and a loop adjacent to the active site residues of another monomer. This β3/β4 loop interfaces with a distinct dynamic α1/β2 loop of another monomer at the interphase between monomers. The C-terminal region of subunit A is highly correlated to the active site residue D37 via the highly fluctuating β3/β4 loop region of subunit C that lies between the two areas. The active site residue R73, located just after β4, is also highly correlated to the C-terminal region because of its proximity (Figure 5C).

4-OT stands out due to its deviation from being a homotrimer. For comparative analysis with the other TSF proteins, a dimer of 4-OT is regarded as a pseudo-monomer and thus, contains two C-terminal segments. Focusing on the catalytically relevant residues located on monomer A, we observe that the C-terminal region of monomer A at K59 demonstrates intra-subunit correlations to the active site residue F50, which is located at the beginning of the β3 strand (Figure 5D). Besides the intra-subunit correlations, the C-terminal region of a given monomer was found to form inter-subunit correlations with active site residues from adjacent monomers. For example, S58 derived from the C-terminal region of monomer C communicates with the active site residue R39 of monomer A, via P56 and I52 of monomer C (Figure 5D).

These correlation analyses have revealed robust communication between the C-terminal residues and crucial active site residues in every TSF protein included in this study. Through these correlated motions, the C-terminal region dynamically influences active site residues and strongly suggests that their impact in catalysis should be further explored with kinetic assays, utilizing C-terminal variants.

## 3. Discussion and Conclusions

The correlation between protein dynamics and functionality in the TSF is largely unknown with the exception of the two human proteins MIF and D-DT. Previous studies on these proteins highlighted the critical role of correlated motions in the regulation of their functionality, despite the low conformational flexibility of their biological assemblies [7,16,34]. In an effort to enhance understanding of structure–activity relationships in the TSF, we analyzed triplicate 1 μs MD simulations of the four bacterial proteins cis-CaaD, CHMI, MSAD, and 4-OT in comparison to MD simulations of the well-studied MIF and D-DT as points of reference.

Despite being members of the same superfamily, our findings demonstrate striking distinctions between the human and bacterial proteins. First, the four bacterial proteins have one region of high flexibility that is absent from MIF and D-DT. In cis-CaaD, CHMI, and 4-OT, this region is the C-terminus, while in MSAD, it is the β3/β4 loop. Another shared feature of the bacterial proteins that is not present in the two human proteins is the formation of long-range inter-subunit communication expanding across their biological assembly. A representative example includes the three T34 residues of cis-CaaD, which are located ~34 Å apart in the biological assembly. Such regions, which are clearly marked on the correlation plots (3× for cis-CaaD, 2× for CHMI, 2× for MSAD, and 1× for 4-OT), possess a mechanistic value aiding efforts to understand the functionality of these proteins. In cis-CaaD, for example, it suggests that conformational changes in T34 accompanied by movements of the C-terminal region are required for substrate binding.

A consistent long-range inter-subunit correlation between the C-termini of cis-CaaD, CHMI, MSAD, and 4-OT led to further investigation of its potential mechanistic value. Our analysis shows that active site residues and the C-terminal region are highly correlated implying a functional role of the latter in catalysis. While our findings provide insights into the correlation between protein dynamics and functionality, this study is limited by the constraints of computational analyses. Future studies including experimental validation, would further clarify the role of C-terminal flexibility in the functionality of these proteins. This could be achieved via site-directed mutagenesis of the C-terminal region for the generation of protein variants with novel structural properties. Each variant would be characterized via protein crystallography, biophysical, and biochemical assays providing insights into the structure-function relationships. Notably, similar experimental analyses of MIF [36] and D-DT [17] showed that the C-terminal region is linked with the catalytic activities of these proteins.

In summary, this study reveals the dynamic maps of cis-CaaD, CHMI, MSAD, and 4-OT through which novel findings with potential mechanistic value were extracted. Such findings promote an understanding of TSF functionalities and provide the foundation for further studies focusing on structure–activity relationships of this intriguing superfamily.

## 4. Materials and Methods

### 4.1. Multiple Sequence Alignments

The FASTA sequences of MIF (PDB:3DJH), D-DT (PDB:1DPT), cis-CaaD (PDB:2FLZ), CHMI (PDB:3E6Q), MSAD (PDB:2AAG), and 4-OT (PDB:1OTF) were obtained from the corresponding deposited crystal structures. In the case of CHMI, the N-terminus tag was removed prior to the alignment. MSA alignment of the six proteins was generated using the standalone program ESPript_3.0 [42]. The core input files were pre-aligned in Clustal, FASTA, MultAlin, NPS@, or ProDom (https://www.ebi.ac.uk/jdispatcher/msa/clustalo, accessed on 28 October 2024) format. The query file for the EsPript run was obtained from Clustal Omega [43]. EsPript then calculates a similarity score for each residue in the aligned sequences. A red box and a white character designate strict identity. The red characters designate similarity in a group. The blue frame designates similarity across groups. The default global score of 0.7 was changed to 0.6. If the similarity score assigned to a column was greater than 0.6, then the corresponding residues in the column were marked as highly similar.

### 4.2. MD Simulations

Generation of simulation data was carried out on Linux machines utilizing, where applicable, CUDA accelerated or parallelized versions of simulation, analysis, and modeling programs. Starting structures for the 1 µs simulations were obtained from the Research Collaboratory for Structural Bioinformatics (RCSB) Protein Data Bank for the corresponding proteins: 3DJH MIF, 1DPT D-DT, 1OTF 4-OT, 2FLZ cis-CaaD, 2AAG MSAD, and 3E6Q CHMI. Where necessary, symmetry pairs of the deposited structure were generated to obtain the biological assembly using the PyMOL Molecular Graphics System, Version 2.0 Schrodinger, Inc. [44]. Crystallographic waters, ions, and small molecules were removed. Missing residues were added using UCSF Chimera [45]. Protein structure files (PSF) with hydrogens were generated using the Visual Molecular Dynamics (VMD 1.9.3) [46] plugins psfgen 1.6.4. and CHARMM36 topology [47]. The resulting PSF and PDB pair were then solvated with TIP3P waters, and the net-charge of the system was checked using the VMD solvate (1.7) and autoionize (1.4) plugins, respectively [46]. Waters were added based upon the dimensions of the protein of interest with a minimum of 7.5 Å of waters bordering the perimeter of the structure. Where necessary, the charge of the system was balanced with Na^+^ or Cl^−^ ions. The simulation process was carried out in a similar manner as previously described [15] using NAMD 2.12 [48], with a 2 fs timestep. Throughout the entire simulation process, the non-bonded interaction distance cutoff was set to 12 Å. Periodic boundary conditions were also used and set to the size of the system, while Langevin dynamics were used to simulate the system’s temperature. Minimization of the system was conducted utilizing a multistep process. First, the protein was fixed in the system and only the solvent was minimized. Next, minimization of the system was conducted with the backbone of the protein fixed. Finally, the entire system was minimized, before a gradual heating to 300 K. The system was then equilibrated for 1 ns at 300 K before a production run of 1ms was performed with all outputs being generated every 10 ps. All minimization, equilibration, and production runs were completed in triplicate from the solvated and charge balanced structures.

### 4.3. Correlation Analysis

Generalized cross-correlation analysis was performed using the Cα over the 1 µs trajectory utilizing the GROMACS plug-in g_correlation (version 1.0.3) [49]. The correlation values produced from this analysis ranged between 0 and 1 (where 0 means no correlation and 1 means absolute correlation) and relate the movements of different Cα with each other. A correlation value of 1 is expected when comparing a group against itself and high values nearing 1 are typical when comparing neighboring groups. High correlation values that appear between non-neighboring groups can provide a further understanding of important intra and inter-subunit interactions and elucidate changes in dynamics and communication that occur when an inhibitor is bound.

### 4.4. RMSF Analysis

RMSF analysis of Cα was performed in GROMACS by using the coordinates of the first frame of the production MD run and the trajectory in GROMACS TRR format. RMSF analysis was performed for each triplicate and RMSF values were averaged.

## Data Availability

All data is provided and analyzed within the manuscript.

## Supplementary Materials

The following supporting information can be downloaded at https://www.mdpi.com/article/10.3390/ijms252312617/s1.
