# Supplementary material for: Conformational Flexibility of the C-Terminal Region Influences Distal Active Site Residues Across the Tautomerase Superfamily
Source: Int J Mol Sci. 2024 Nov 24;25(23):12617. doi: 10.3390/ijms252312617 (PMC11641302; doi:10.3390/ijms252312617)
Supplement: Supplementary file 1 [file ijms-25-12617-s001.zip › ijms-3318821-supplementary.pdf]

SUPPLEMENTAL MATERIAL

Conformational Flexibility of the C-Terminal Region Influences Distal Active Site Residues Across the Tautomerase Superfamily

Christopher Argueta[†] , Andrew Parkins[†], and Georgios Pantouris[*]

Department of Chemistry, University of the Pacific, Stockton, CA 95211, USA

[†]Equal contributing authors

[*]**Corresponding author**: gpantouris@pacific.edu

Table S1: Sequence identity between TSF members*

	MIF	D-DT	cis-CaaD	CHMI	MSAD	4-OT
**MIF**	-	34.2	16.5	15.7	19.1	14.6
**D-DT**	34.2	-	12.8	21.9	19.5	16.4
**cis-CaaD**	16.5	12.8	-	22.9	25.0	14.3
**CHMI**	15.7	21.9	22.9	-	24.7	16.0
**MSAD**	19.	19.5	25.0	24.7	-	25.8
**4-OT**	14.6	16.4	14.3	16.0	25.8	-

*The numbers are expressed in percent (%) identity

Table S2: Average Root Mean Square fluctuation (RMSF) profiles of TSF proteins*

	Average RMSF (Å)
**MIF**	0.71 ± 0.2
**D-DT**	0.69 ± 0.3
**cis-CaaD**	0.88 ± 0.7
**CHMI**	0.67 ± 0.5
**MSAD**	0.79 ± 0.5
**4-OT**	0.87 ± 0.7

*Data were obtained from three independent 1μs MD simulations

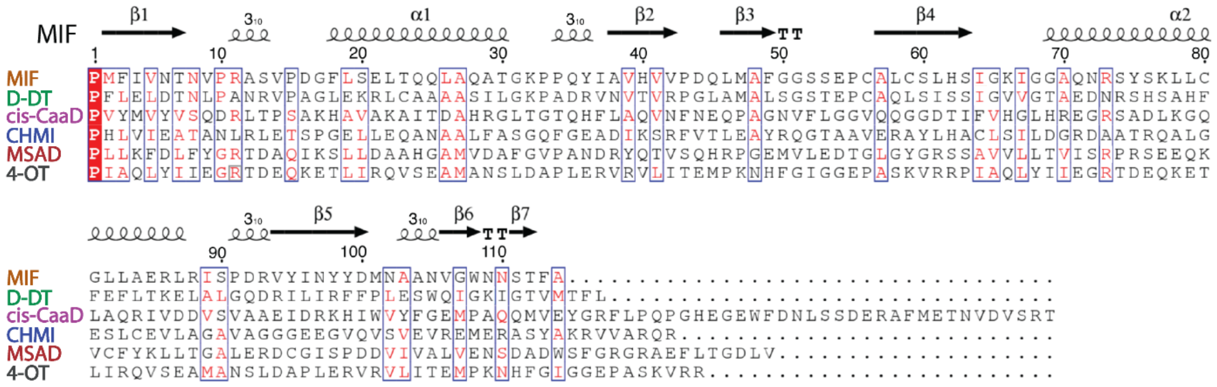

Figure S1. **Multiple sequence alignment of TSF representative members**. The amino acid sequences of human MIF (PDB:3DJH), human D-DT (PDB:1DPT), *Coryneform bacterium* cis-CaaD (PDB:2FLZ), *Pseudomonas aeruginosa* CHMI (PDB:3E6Q), *Pseudomonas pavonaceae* MSAD (PDB:2AAG), and *Pseudomonas sp. CF600* 4-OT (PDB:1OTF) were obtained from RCSB PDB (protein data bank) using the corresponding entries shown in each parenthesis. A red box and a white character (catalytic residue P1) designates strict identity. The red characters designate similarity in a group. The blue frame designates similarity across groups. For comparison, the secondary structure of MIF is provided on the top of the panel. Multiple sequence alignment was performed using Clustal Omega [1].

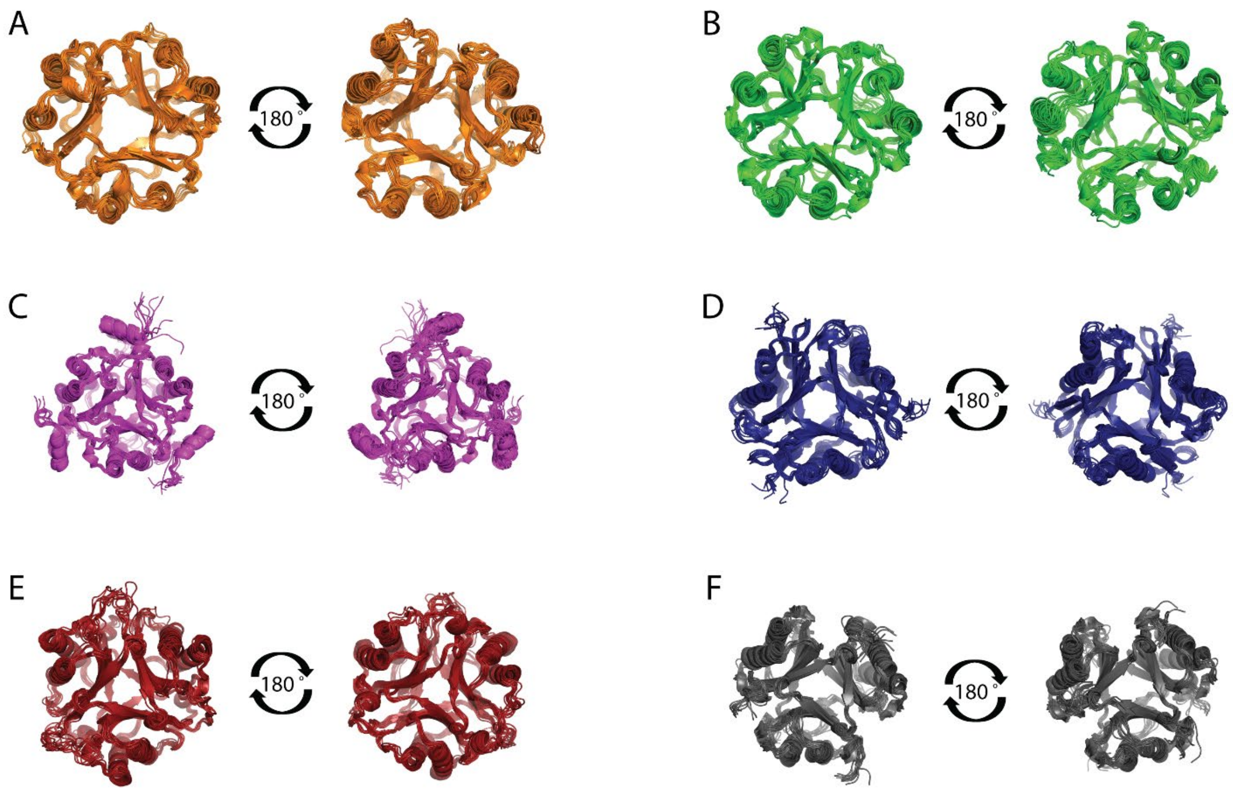

Figure S2. **Dynamic profiles of the six TSF proteins**. Snapshots of **A)** MIF, **B)** D-DT, **C)** cis-CaaD, **D)** CHMI, **E)** MSAD, and **F)** 4-OT structures were captured at 100 ns increments, having 0 ns and 1μs as the starting and ending points, respectively. For accuracy, the one microsecond trajectories were repeated in triplicate.

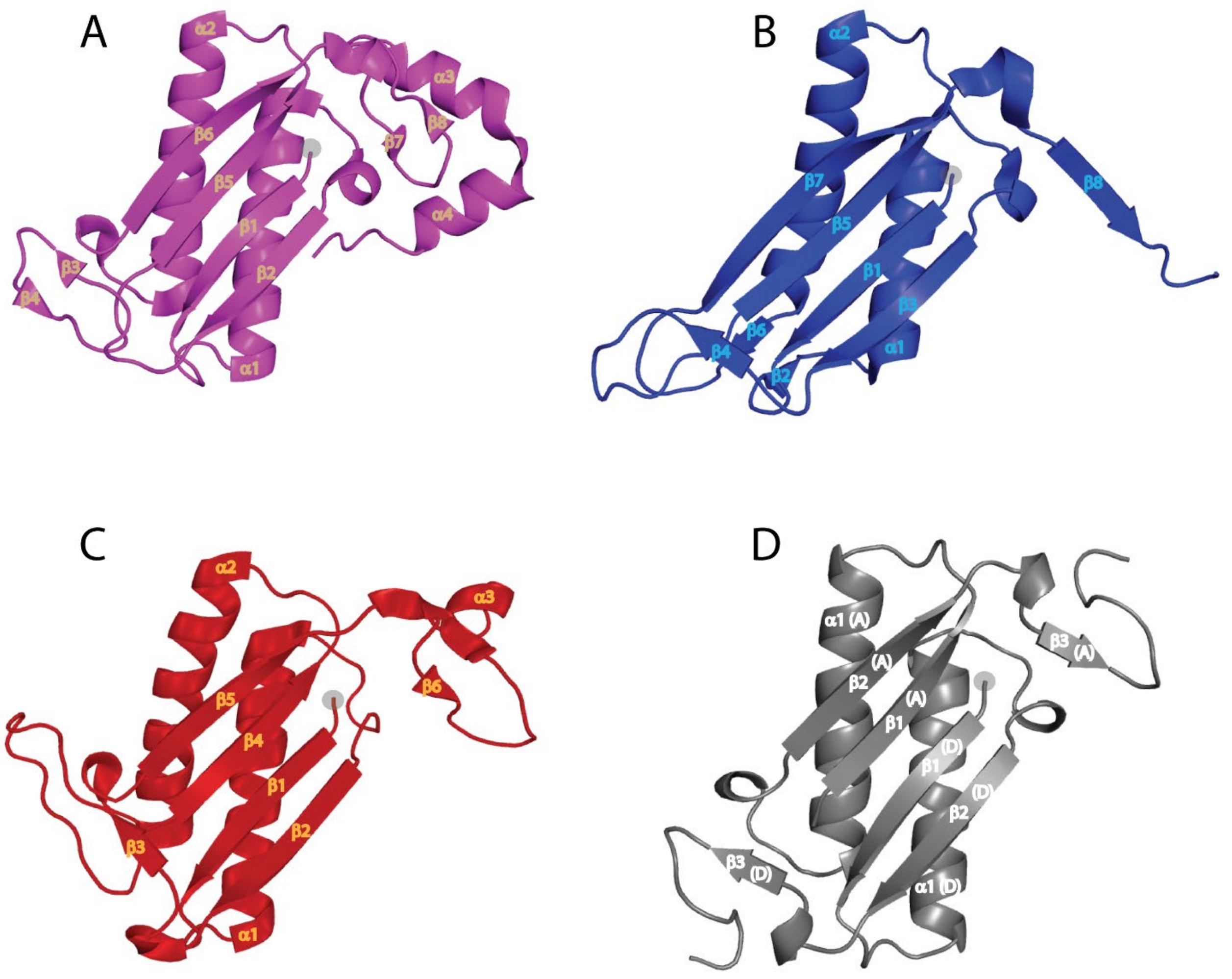

Figure S3. **The secondary structure features of the four bacterial proteins.** The **(A)** cis-CaaD (PDB: 2FLZ), **(B)** CHMI (PDB: 3E6Q), **(C)** MSAD (PDB: 2AAG) monomers and **(D)** 4-OT (PDB: 1OTF) dimer were used for this analysis. The position of P1 is highlighted with a transparent sphere.

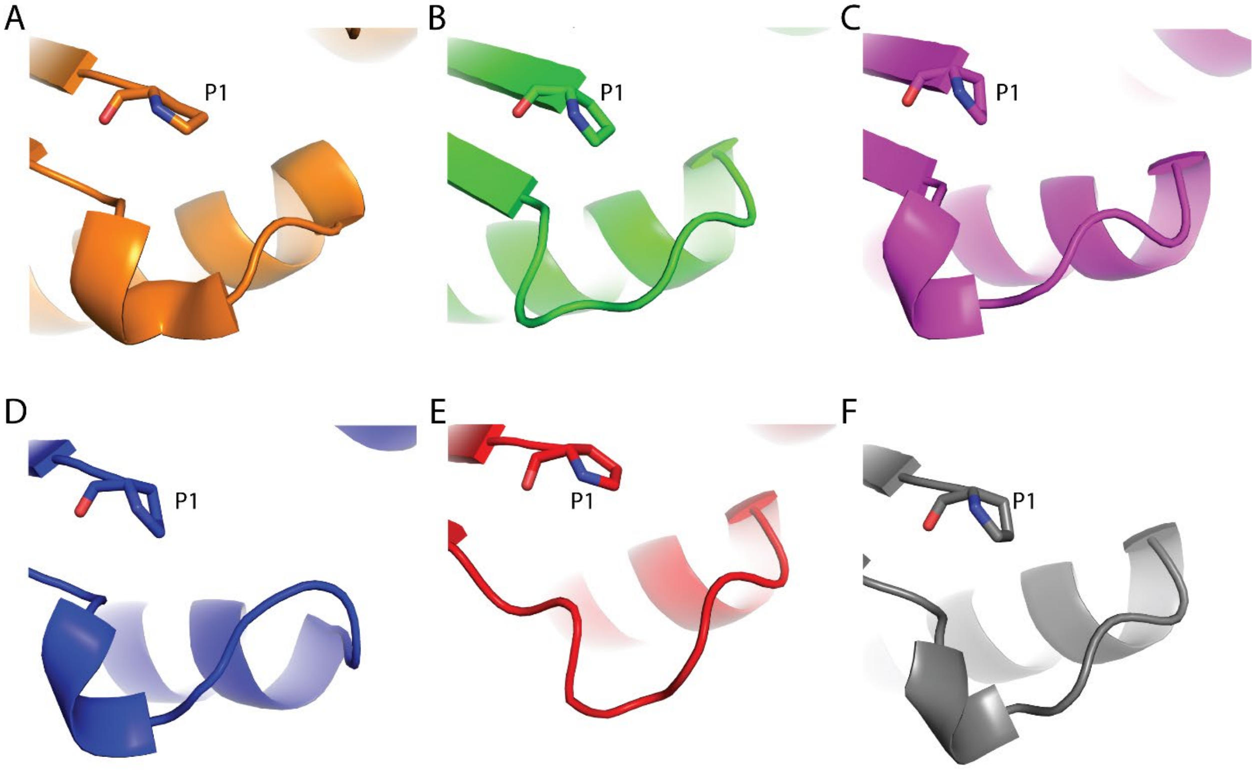

Figure S4. **Illustration of the flexible loop, found in the active site environment of the six TSF proteins.** This α1/β2 loop (the corresponding α1/β3 loop for CHMI) is located next to the catalytic residue P1. The six illustrations were derived from the crystal structures of **A)** MIF (PDB:3DJH), **B)** D-DT (PDB:1DPT), **C)** cis-CaaD (PDB:2FLZ), **D)** CHMI (PDB:3E6Q), **E)** MSAD (PDB:2AAG), and **F)** 4-OT (PDB:1OTF). The catalytic residue P1 is shown as sticks.

REFERENCES

1. Sievers, F.; Wilm, A.; Dineen, D.; Gibson, T. J.; Karplus, K.; Li, W.; Lopez, R.; McWilliam, H.; Remmert, M.; Soding, J.; Thompson, J. D.; Higgins, D. G., Fast, scalable generation of high-quality protein multiple sequence alignments using Clustal Omega. *Mol Syst Biol* **2011,** 7, 539.
